# Supplementary material for: Metagenomic estimation of absolute bacterial biomass in the mammalian gut through host-derived read normalization
Source: mSystems. 2025 Jul 31;10(8):e00984-25. doi: 10.1128/msystems.00984-25 (PMC12363224; doi:10.1128/msystems.00984-25)
Supplement: Supplemental information — Fig. S1 and Table S1. [file msystems.00984-25-s0001.docx]

**Supplementary Information**

**
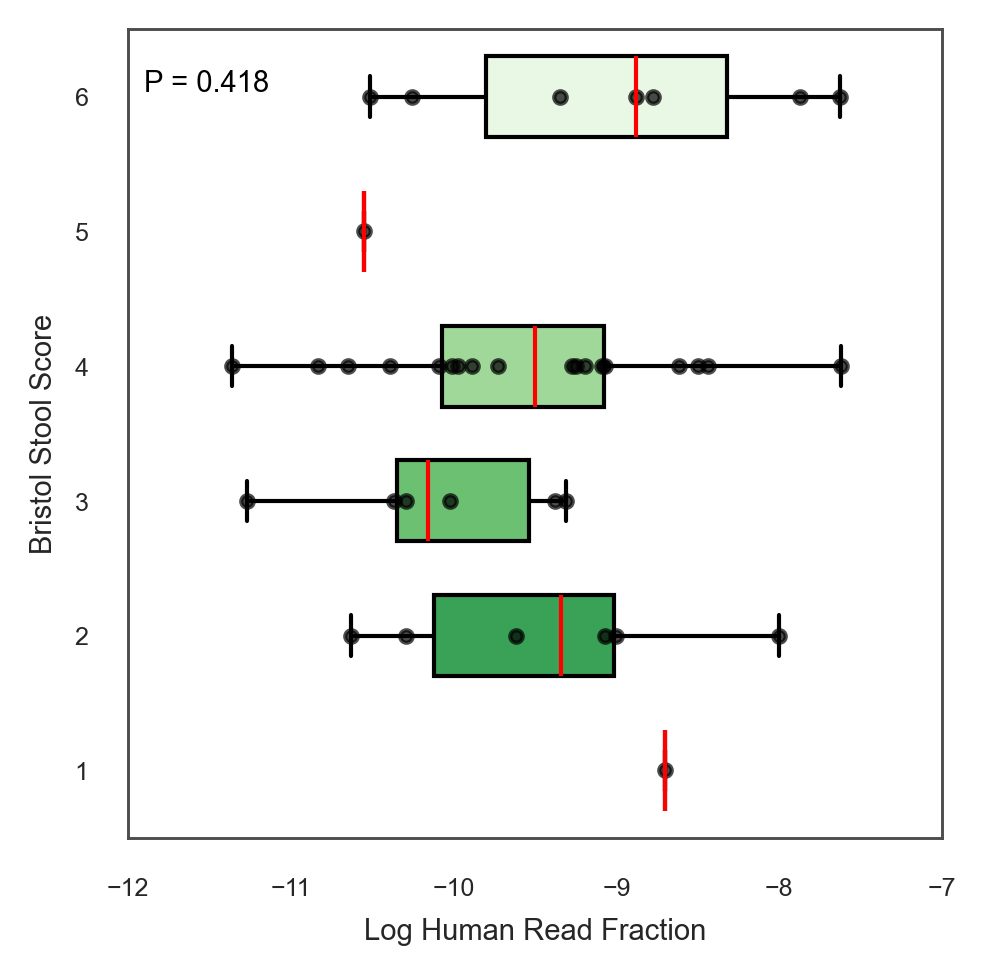
**

**Figure S1. Distribution of log-transformed human read fractions across different Bristol Stool Scores.** Boxplots showing human read fractions (relative to total metagenic reads) across Bristol stool score categories (n = 39). Each boxplot displays the center line (median), box limits (first and third quartiles), and whiskers (1.5 × interquartile range). Using ordinal logistic regression, we did not observe a significant association between human read fractions and Bristol scores (P=0.418).

| **Variable** | **Coefficient** | **Std Err** | **t-value** | **p-value** | **95% CI** |
| --- | --- | --- | --- | --- | --- |
| **B:H ratio ~ covariates** | | | | | |
| **R^2^** = 0.063, **Adjusted-R^2^** = 0.041, **n** = 705 | | | | | |
| Intercept  Mapped read count  Age (years)  BMI (kg/m2)  Waist circumference (cm)  Fasting plasma triglycerides (mmol/L)  Fasting plasma adiponectin (mg/L)  Fasting plasma CRP (mg/L)  HbA1c (%)  Systolic blood pressure (mmHg)  Diastolic blood pressure (mmHg)  Left ventricular ejection fraction (%)  Fasting plasma pro-ANP (pmol/L)  Physical activity (h/week)  Gender  Nationality  Diabetic status | 6.6576  2.577×10^8^  0.0106  -0.0090  0.0191  -0.1271  -0.0415  -0.0040  -0.0759  -0.0033  -0.0033  -0.0085  -1.917×10^-8^  0.0164  -0.0123  -0.1544  0.0252 | 1.005  1.5×10^8^  0.006  0.019  0.008  0.054  0.021  0.008  0.064  0.004  0.006  0.006  0.000  0.009  0.138  0.137  0.145 | 6.625  1.711  1.802  -0.464  2.419  -2.356  -1.982  -0.516  -1.191  -0.816  -0.536  -1.536  -0.152  1.760  -0.089  -1.125  0.174 | 0.000  0.088  0.072  0.643  **0.016**  **0.019**  **0.048**  0.606  0.234  0.415  0.592  0.125  0.879  0.079  0.929  0.261  0.862 | 4.685 – 8.631  3.8×10^-9^– 5.53×10^8^  -0.001 – 0.022  -0.047 – 0.029  0.004 – 0.035  -0.233 – -0.021  -0.083 – -0.000  -0.019 – 0.011  -0.201 – 0.049  -0.011 – 0.005  -0.015 – 0.009  -0.019 – 0.002  -0.000 – 0.000  -0.002 – 0.035  -0.283 – 0.258  -0.424 – 0.115  -0.260 – 0.310 |
|  | | | | | |
| **Microbial load ~ covariates** | | | | | |
| **R^2^** = 0.055, **Adjusted-R^2^** = 0.033, **n** = 693 | | | | | |
| Intercept  Mapped read count  Age (years)  BMI (kg/m2)  Waist circumference (cm)  Fasting plasma triglycerides (mmol/L)  Fasting plasma adiponectin (mg/L)  Fasting plasma CRP (mg/L)  HbA1c (%)  Systolic blood pressure (mmHg)  Diastolic blood pressure (mmHg)  Left ventricular ejection fraction (%)  Fasting plasma pro-ANP (pmol/L)  Physical activity (h/week)  Gender  Nationality  Diabetic status | 2.827×10^11^  -1906.6495  -1.205×10^8^  -5.933×10^8^  1.146×10^7^  -6.801×10^9^  -8.879×10^8^  -1.472×10^8^  -5.783×10^9^  4.069×10^7^  2.213×10^6^  -5.619×10^8^  3.135×10^6^  -6.041×10^8^  5.757×10^9^  -2.112×10^10^  -1.536×10^9^ | 4.78×10^10^  695.958  2.74×10^8^  8.96×10^8^  3.64×10^8^  2.52×10^9^  9.76×10^8^  3.57×10^8^  2.98×10^9^  1.86×10^8^  2.89×10^8^  2.63×10^8^  5.87×10^6^  4.35×10^6^  6.43×10^9^  6.45×10^9^  6.86×10^9^ | 5.918  -2.740  -0.440  -0.662  0.031  -2.703  -0.910  -0.413  -1.939  0.219  0.008  -2.135  0.534  -1.390  0.896  -3.272  -0.224 | 0.000  **0.006**  0.660  0.508  0.975  **0.007**  0.363  0.680  0.053  0.827  0.994  **0.033**  0.593  0.165  0.371  **0.001**  0.823 | 1.89×10^11^ – 3.76×10^11^  -3273.148 – -540.151  -6.59×10^8^ – 4.18×10^8^  -2.35×10^9^ – 1.17×10^9^  -7.04×10^8^ – 7.27×10^8^  -1.17×10^10^ – -1.86×10^10^  -2.8×10^9^ – 1.03×10^9^  -8.48×10^8^ – 5.53×10^8^  -1.16×10^10^ – 7.18×10^7^  -3.24×10^8^ – 4.05×10^8^  -5.66×10^8^ – 5.7×10^8^  -1.08×10^9^ – -4.51×10^7^  -8.39×10^6^ – 1.47×10^7^  -1.46×10^9^  – 2.49×10^8^  -6.86×10^9^ – 1.84×10^10^  -3.38×10^10^ – -8.44×10^9^  -1.5×10^10^ – 1.19×10^10^ |

**Table S1. Multivariable Linear Regression Models Evaluating Associations Between B:H Ratio, Microbial Load, and Relevant Covariates in the MetaCardis Cohort.** Results from multiple linear regression models assessing the association between covariates and two key outcomes: the log-transformed B:H read ratio and cytometric microbial load. Statistically significant coefficient p-values (p < 0.05) are shown in bold.
